# Predicting Fluorescence Emission Wavelengths and Quantum Yields via Machine Learning

**DOI:** 10.1021/acs.jcim.4c02403

**Published:** 2025-03-20

**Authors:** Rubens C. Souza, Julio C. Duarte, Ronaldo R. Goldschmidt, Itamar Borges

**Affiliations:** † Departamento de Engenharia de Defesa, Instituto Militar de Engenharia (IME), Praça Gen. Tibúrcio 80, Rio de Janeiro, Rio de Janeiro 22290 270, Brazil; ‡ Departamento de Engenharia da Computação, Instituto Militar de Engenharia (IME), Praça Gen. Tibúrcio 80, Rio de Janeiro, Rio de Janeiro 22290 270, Brazil; § Departamento de Química, Instituto Militar de Engenharia (IME), Praça Gen. Tibúrcio 80, Rio de Janeiro, Rio de Janeiro 22290 270, Brazil

## Abstract

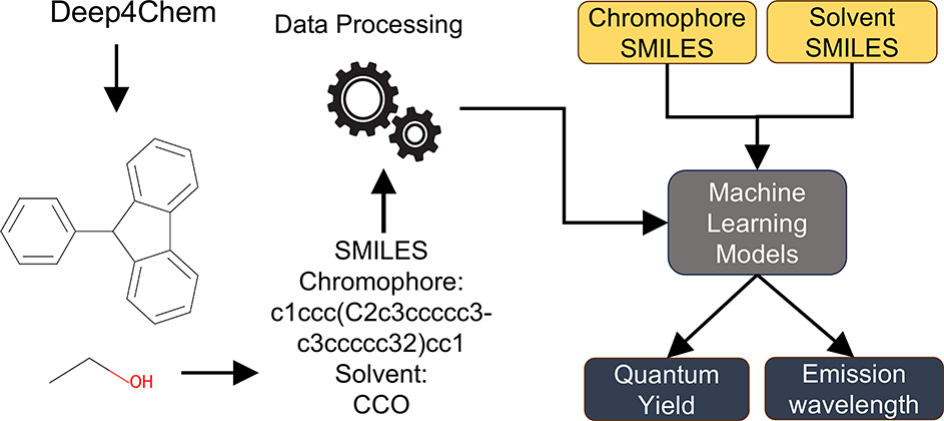

The search for functional fluorescent organic materials can significantly benefit from the rapid and accurate predictions of photophysical properties. However, screening large numbers of potential fluorophore molecules in different solvents faces limitations of quantum mechanical calculations and experimental measurements. In this work, we develop machine learning (ML) algorithms for predicting the fluorescence of a molecule, focusing on two target properties: emission wavelengths (WLs) and quantum yields (QYs). For this purpose, we employ the Deep4Chem database which contains the optical properties of 20,236 combinations of 7,016 chromophores in 365 different solvents. Several chemical descriptors, or features, were selected as inputs for each model, and each molecule was characterized by its SMILES fingerprint. The Shapley additive explanations (SHAP) technique was used to rationalize the results, showing that the most impactful properties are chromophore-related, as expected from chemical intuition. For the best-performing model, the Random Forest, our results for the test set show a root-mean-square error (RMSE) of 28.8 nm (0.15 eV) for WLs and 0.19 for QYs. The developed ML models were used to predict, thus completing, the missing results for the WL and QY target properties in the original Deep4Chem database, resulting in two new databases: one for each property. Testing our ML models for each target property in molecules not included in the original Deep4Chem database gave good results.

## Introduction

The rationalization and development of fluorescent organic molecules, probes, or fluorophores are essential from a fundamental perspective because they involve basic processes^1^ and are crucial components of various applications.^2^ For example, in medicine, hydrophobic fluorescent probes have been widely used in studies of model cell membranes.^3^ In physics and materials science, these molecules have been employed in developing electrically pumped organic lasers,^4^ stimulated emission depletion microscopy,^5^ thermally activated delayed fluorescence organic light-emitting diodes,^6^ and different fluorescent chemical sensors.^7^ Therefore, discovering new fluorescent substances can open new possibilities for biological and chemical applications.^8^ As the discovery of new fluorescent materials depends on identifying electronic properties related to emission wavelengths and quantum yields,^9^ it is essential to develop new approaches to accelerate this process.

The emission wavelength (WL) is a critical property for practical applications of fluorophores^10^ because it determines the selection of molecules that emit light within the desired spectral range. Similarly, the quantum yield (QY) measures the efficiency with which a fluorophore converts absorbed energy into emitted light; thus, it is essential for maximizing light emission and minimizing energy loss. Therefore, the accurate prediction of these electronic photophysical properties is crucial for developing highly efficient and specific fluorophores for various applications.

New fluorophores are typically developed by modifying existing structures.^8^ While accurate quantum chemical methods are available to predict molecular properties, the thorough theoretical screening of potential candidates is computationally demanding.^11^ Moreover, even after identifying promising molecules through computational approaches, their synthesis and experimental characterization require substantial time and financial resources.^12^

Many studies have combined chemical data with machine learning (ML) techniques to overcome the challenge of obtaining accurate results and minimizing the computational costs of computing emission wavelengths in molecules.^13−18^ Other investigations of photochemical properties that combine ML and molecular databases include the study of orbitals and electronically excited states.^19−24^ Our group has employed ML techniques to study the impact sensitivity of energetic molecules,^25^ establish a consistent new set of Hammett constants,^26^ explore materials for organic electronic device applications,^26^ investigate the thermophysical properties of fluids across various thermodynamic phases,^27,28^ and predict electronically excited state molecular properties.^29^

Ju and collaborators^18^ also used ML techniques to investigate solvated organic fluorescent dyes’ WLs and photoluminescence QYs. They employed a database containing 3000 distinct compounds and used molecular fingerprints as input features for their ML models. The best ML model was the gradient boosting regression tree (GBRT), which had an accuracy comparable to that of computational quantum calculations. This ML approach could potentially lead to significant time savings in screening fluorescent molecules.

Joung and collaborators^30^ built the Deep4Chem database of the experimental properties of molecules by collecting data from various experimental studies; the authors developed deep learning models to predict key optical properties, including absorption peak position, absorption bandwidth, extinction coefficient, emission peak position, emission bandwidth, photoluminescence quantum yield, and emission lifetime. Their ML model was a graph convolutional network (GCN) trained using matrices incorporating chromophore atom connectivity information. Specifically, the feature matrix includes details about identity, the number of hydrogen and heavy atoms bonded to them, aromaticity, hybridization, ring structure, and formal charge.^31^ Sun and coworkers^32^ used the same database^30^ to build ML models to predict key properties such as absorption and emission wavelengths, photoluminescence quantum yields, and full width at half-maximum. Structural features of the molecules were employed as input for models based on a deep learning framework, specifically, a classic message-passing neural network (MPNN). This approach effectively captures the structural features of optical properties, even when working with a limited dataset.

Although many databases are made available every year, experimental databases such as Deep4Chem^30^ do not provide all properties for every molecule, often making the WLs and QYs available for some molecules but not others. This database was selected for this work to evaluate the fluorescence efficiency of molecules, focusing on creating ML models for WLs and QYs and filling in the data gaps. Our primary interest here was in identifying the main chemical properties that contribute to predicting these two target properties for the molecules in Deep4Chem.^30^ We also rationalized the fluorescence phenomenon by employing Shapley additive explanations (SHAP)^33^ plots.

## Methods

As input for the ML models, we use the Simplified Molecular Input Line Entry System (SMILES)^34^ format from the Deep4Chem database^30^ to represent a molecular structure and generate chemical descriptors with the RDKit^35^ tool. Our ML models implement techniques and tools such as LazyPredict,^36^ cross-validation,^37^ and hyperparameter testing.^38^ The generated ML models are then used to fill in the missing data in the original Deep4Chem database.

The Deep4Chem^30^ database contains molecules in the SMILES^34,39^ format with well-defined experimental properties in a CSV-format file. The distribution of the WL and QY values in Deep4Chem is depicted in Figure S1. This database is a comprehensive compilation of the optical experimental properties of organic chromophores developed to support data-driven research in fields such as optoelectronics and bioimaging. It aggregates 20,236 data points (i.e., molecules and their properties) from 1,358 peer-reviewed articles, documenting the experimental absorption and emission spectra of 7,016 unique chromophores in various solvents and in the solid state. Key properties include maximum emission wavelengths (λ_emi,max_, labeled here as WLs) and photoluminescence quantum yields (QYs). The database provides emission wavelengths spanning from the UV to near-infrared, thus providing resources to assist in the design of dyes, sensors, and light-emitting diodes. The QY values, carefully selected from degassed conditions where possible, represent the efficiency of photoluminescence processes, making this resource relevant for developing materials with optimized optical performance.

The RDKit tool was developed to generate chemical descriptors (fingerprints), digital representations or descriptors that concisely and numerically capture a molecule’s structural, physicochemical, or other properties.^35^ We used RDKit and Python to extract molecular properties used as inputs in our ML models. RDKit requires the molecule’s SMILES format to generate molecular descriptors and fingerprints.

The ML approaches developed in this work were implemented using the Scikit-learn library (scikit-learn-1.4.0)^40^ in Python.^41^ Molecular fingerprints were obtained from RDKit,^35^ including the Morgan fingerprints with the GetMorganFingerprintAsBitVect function and the MACCS keys with the GetMACCSKeysFingerprint function. These functions generate binary vector representations of molecules that capture information about their chemical structure. A 512-bit vector was chosen for the Morgan fingerprints to balance specificity and generality in representing the substructures. By default, MACCS fingerprints use a 167-bit vector. Detailed descriptions of these fingerprints can be found in Section S2. Additionally, 54 other chemical descriptors were selected, as detailed in Table S1.

Before training any ML model, descriptors are generated for all molecules in the dataset. Using RDKit, molecular descriptors are extracted from SMILES strings and organized into a feature matrix ***X***. The corresponding target properties are stored in a separate vector ***y***. To ensure data quality, entries with missing target properties or extreme outlier values (i.e., values significantly deviating from the dataset distribution) are excluded from the analysis. To finalize data cleaning, the ***X*** set of descriptors, independent for each model – ***X***_1_ for WLs and ***X***_2_ for QYs – is normalized using the StandardScaler tool from Scikit-learn. Specifically, among the 20,236 molecule–solvent combinations, 18,142 include the WL property, while 13,837 include the QY property.

**Figure 1:**
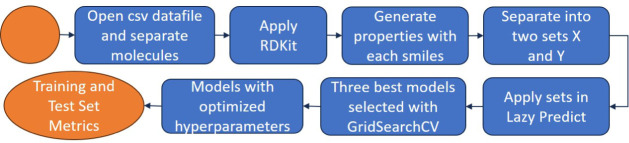
Methodology for building machine learning models.

Figure 1 depicts the proposed methodology employed in this work. The approach is similar to the one we used in a previous investigation.^29^

**Figure 2:**
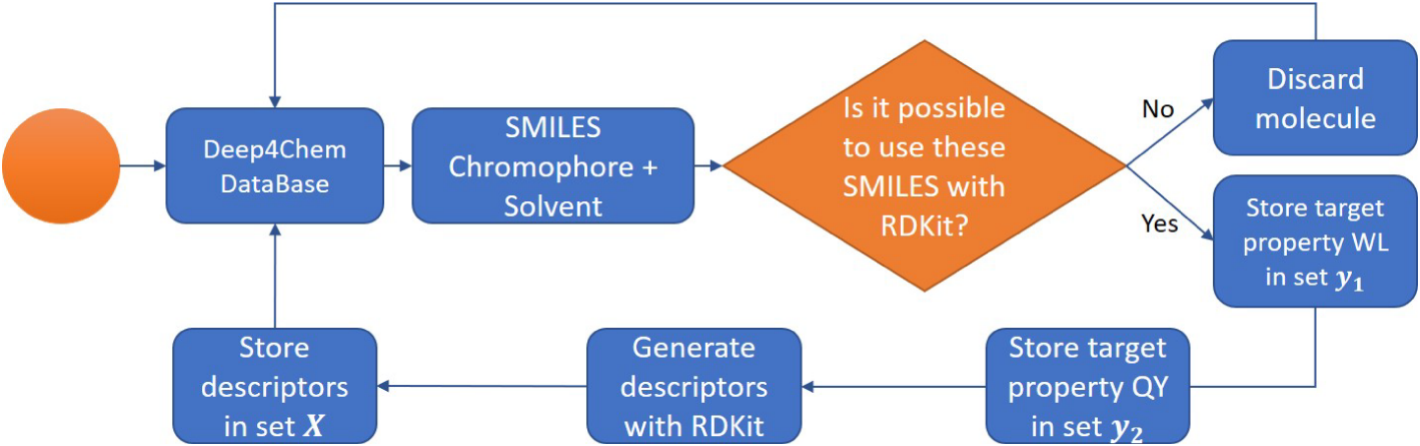
Storage stream of the sets built from the original Deep4Chem^30^ includes emission wavelengths (WLs) and quantum yields (QYs).

All training sets for each molecule represented by a given SMILES string include the same descriptors and the input properties for each molecule obtained from RDKit. In our case, the target properties are represented by a vector ***y***_1_ for the WLs and ***y***_2_ for the QYs. The input matrix ***X*** consists of 1,466 columns (512 Morgan “chromophore”, 512 Morgan “solvent”, 167 MACCs “chromophore”, 167 MACCs “solvent”, 54 chemical descriptors “chromophore”, and 54 chemical descriptors “solvent”), with 733 descriptors for each SMILES (i.e., for each molecule). Figure 2 shows the workflow for storing the molecules and their properties in our database, which was developed from the original Deep4Chem database.

A preliminary screening of the best ML models for our problem employed the Lazy Predict tool from Scikit-learn, which quantifies uncertainties through specific error metrics. We tested using Lazy Predict 41 ML models for the WL and QY target properties by employing the RDKit input parameters. This process was repeated twice (once for WLs and once for QYs), and the results are presented in Tables S2 and S3. Lazypredict tests all its models using its default parameters without performing hyperparameter optimization. Due to computational limitations, training all models with optimization and cross-validation was impractical. Therefore, the three most frequent ML algorithms appearing among the top ten results for the two target properties were selected. An additional model, an artificial neural network (ANN), was developed for comparison purposes. The ANN model comprises interconnected units called artificial neurons that process information across multiple layers. We used a feedforward neural network of the multilayer perceptron (MLP) type.^42^ Each target property – WL or QY – was investigated by using the four ML algorithms.

In total, 8 ML models (4 for each target property) were trained using the molecules from the database constructed in this work to predict the target properties WL and QY. After the data preprocessing and transformation stage, the dataset did not retain its original size, as some molecules could not be interpreted by RDKit or, in some cases, lacked target properties; in such cases, these molecules were removed from the dataset. This limitation occurs because RDKit relies on strict chemical rules and validation checks, which can reject SMILES strings that are malformed, contain unsupported elements, or violate basic chemical structure and valence principles. Therefore, the ***y***_1_ database for WLs has 18,079 entries, ***y***_2_ for QYs has 13,685 entries, comprising 89% and 65% of the entries from the original Deep4Chem dataset. All algorithms were trained from the two processed datasets with a 70% data split for training and 30% for testing. The test set size was chosen to ensure a sufficient amount of data for testing and to maintain the quality of the results.

The GridSearchCV tool from Scikit-learn was employed to determine the optimal hyperparameters for each algorithm. We used this tool for multiple tests and repeated training sessions to obtain optimized hyperparameters for each model based on evaluation metrics. Following the identification of the best algorithms, cross-validation with 3-fold (i.e., three different splits within the training set) was applied, evaluating the models using the MAE (mean absolute error), MSE (mean squared error), RMSE (root mean squared error), and *R*^2^ (coefficient of determination), as described in the Supporting Information. To balance computational efficiency and model robustness, particularly given the dataset’s size and complexity, 3-fold cross-validation was preferred over the commonly used 10-fold approach in GridSearchCV tests. Although 10-fold cross-validation provides a more detailed evaluation, its significantly higher computational expense and longer training time are disproportionate to the marginal improvements in performance evaluation it may offer, as we showed before.^29^

**Figure 3:**
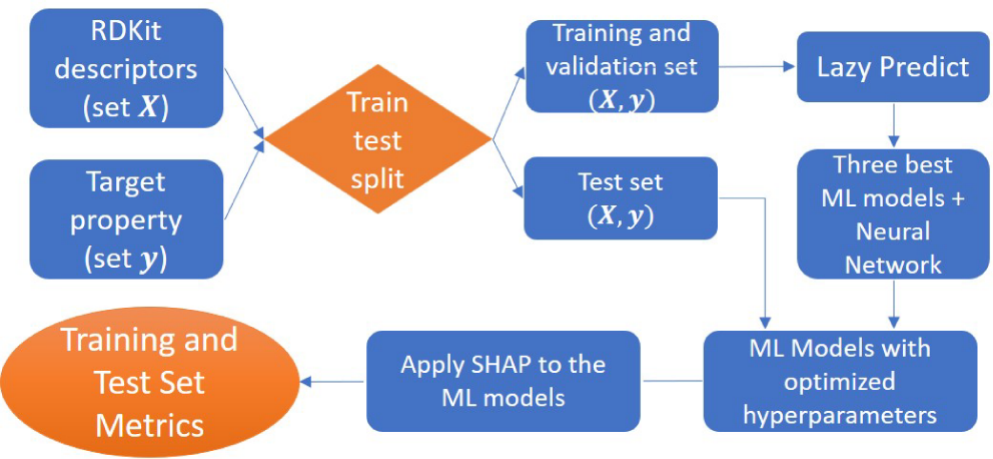
Model explainability process.

Those error metrics were used to evaluate the models because similar values across the training, validation, and test sets indicate good model generalization and a lack of overfitting.^43^ The SHAP^33^ technique was used to assess the impact of each descriptor on the outcome, providing explainability for the results. Figure 3 shows the workflow for evaluating the developed ML models.

As mentioned, not all of the WL and QY values are available for each molecule in the Deep4Chem experimental database. Therefore, the developed ML models were used to predict these missing data points in the original Deep4Chem database. The molecules lacking one or two target properties were isolated and separated, and predictions of these properties were made for each based solely on their SMILES format employing our ML models. As a result, two new databases from the original Deep4Chem were created, each containing SMILES representations of chromophores and solvents and now including predicted values for the previously absent properties. The first database includes the SMILES format for chromophores and solvents and the WL values, both the experimental values from the original Deep4Chem database and those predicted by our ML in the missing cases. The second is similar but now refers to the QY property.

## Results and Discussion

### Error Metrics

The *R*^2^ and RMSE error metrics for the two new datasets with emission wavelength (WL) and quantum yield (QY) values combined with the RDKit chemical descriptors, processed using LazyPredict, are detailed in Tables S2 and S3. The three ML models consistently ranked highest across these metrics: the ExtraTrees, Random Forest (RF), and Extreme Gradient Boosting. Consequently, hyperparameter tuning and cross-validation were conducted for these models and the ANN. Performance metrics, including MAE, MSE, RMSE, and *R*^2^, for the test set of each model are provided in Tables S4–S7. Additional metrics are available in the Python scripts on our GitHub repository and the Zenodo repository,^44^ referenced at the end of this article.

Tables S4–S7 overall show reasonable and similar error metric values for all models and each target property, with the RF model yielding the best test set results for each property. Not even the ANN, despite its modern deep learning architecture, could match the results of RF, and a similar result was found in a previous work.^29^ Therefore, from now on, comparisons and importance analyses will be based on the RF model. For both WL and QY target properties, the RF models were optimized with the following hyperparameters: max_leaf_nodes = 10000, min_samples_split = 7, n_estimators = 100, and random_state = 42. These values are reported here to ensure robust and reproducible results. The 3-fold cross-validation confirmed the results because it displayed similar error metrics; see Tables S8 and S9. The results from the eight models can be found on our GitHub repository and the Zenodo repository.^44^

**Figure 4:**
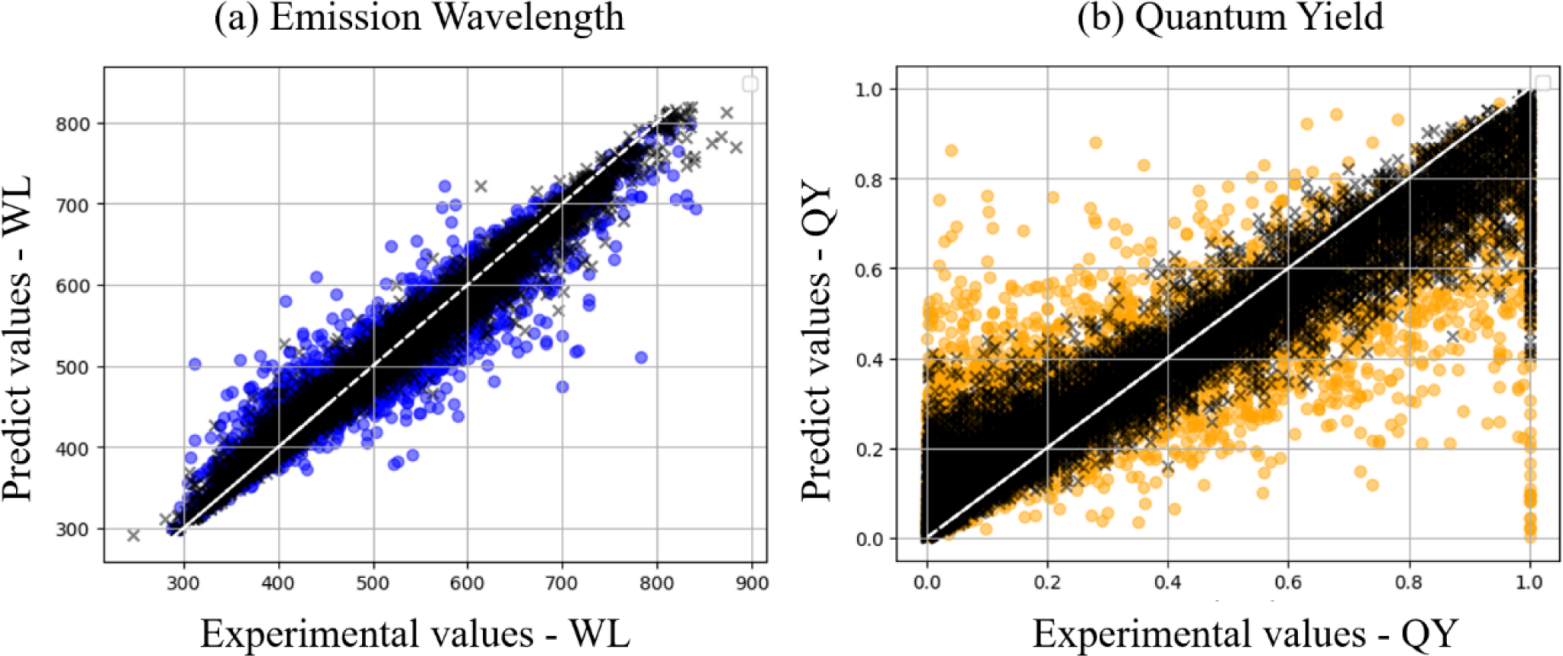
A plot of the Deep4Chem database experimental values and the predicted values (training and test data points) from our Random Forest model. The black points represent the training set predictions, while the colored points (blue for WLs, orange for QYs) correspond to the test set: (a) emission wavelengths (WLs); (b) quantum yields (QYs).

Figure 4 displays the original values from the Deep4Chem dataset alongside the predictions from the RF model applied to both the training and test sets for the WL and QY target properties. The similar metrics observed in each fold, combined with the RMSE learning curves (Figures S2 and S3) and the plots in Figure 4, indicate that the RF model for the WLs and QYs has good convergence. We also carried out additional tests using datasets distinct from our own, presented below, which further confirmed the robustness of the WL and QY models. These additional tests include experimental data and quantum chemistry results. Furthermore, the learning curves (Figures S2 and S3) show that using more experimental data could improve the performance of the ML models.

Analysis of the WL (nm) data distribution (Figure 4a) shows that the model tends to overestimate shorter wavelengths (<500 nm) and underestimate longer ones (>700 nm). This behavior may be related to the uneven data distribution in the training set, as shown in Figure S1a. According to the count of values per interval, most data are concentrated in the 300–700 nm range, with few instances below 300 nm and above 800 nm. This scarcity of data at the extremes may lead to less accurate predictions in these regions, resulting in the observed bias.

The bias is even more pronounced for the QYs. However, this is not only due to the data distribution but also due to the known difficulties in measuring this property.^45^Figure S1b shows a similar distribution for the QYs, starting from values above 0.1. Moreover, the quantum yields are highly dependent on factors such as molecular structure, solvent environment, and energy transfer efficiency, which may not be fully captured by the features used in our model.

We now examine the error metrics for the test set and the RF model for these two properties. For the WLs (Figure 4a), we obtained the following metrics: MAE (18.55 nm/0.09 eV), MSE (828.62 nm/0.02 eV), RMSE (28.78 nm/0.15 eV), and *R*^2^ (0.90 for both). For QYs (Figure 4b), the error metrics for the test set were found to be MAE (0.14), MSE (0.04), RMSE (0.19), and *R*^2^ (0.62).

Our RF model has good generalization and validation set metrics compared to other literature models using the same Deep4Chem database. The GCN model by Joung and coworkers reports an RMSE of 22.5 nm for the WLs (ours: 28.8 nm) and 0.543 (ours: 0.19) for QYs. The difference between the performances of both models can be attributed to both the model architectures and the input features, despite the use of the same database. Although the RF model has a simpler architecture than the GCN model, it still revealed a better performance for the QY target property. Their input for the models consisted of the chromophore structure, represented by a 150 × 150 adjacency matrix and a 150 × 43 feature matrix, including the connectivity information on atoms in the chromophore. The feature matrix in their model identifies the atoms, the number of hydrogen and heavy atoms bonded to them, aromaticity, hybridization, ring structure, and formal charge.

The MPNN model message developed by Sun and coworkers achieves an RMSE of 21.2 nm for WLs (ours: 28.8 nm) and 0.15 (ours: 0.19) for QYs. The input to the MPNN model consisted of molecular graphs of the emitter and solvent, concatenated vertically to better represent chemical characteristics and simplify processing in the model.

To benchmark our ML model against established quantum chemical methods, we identified a recent study from Mandal and coworkers^46^ that systematically compares various TD-DFT/def2-TZVPD approaches for calculating excitation energies. They used the MAE metric in electron volts (eV) to evaluate the model performance, reporting MAE values ranging from 0.19 eV (lowest) to 0.65 eV (highest). We converted our model’s results to eV to enable a direct comparison. We obtained an MAE of 0.096 eV, an MSE of 0.022 eV, and an RMSE of 0.150 eV for the test set, thus demonstrating significantly superior accuracy in this case for predicting (fluorescence) excitation energies.

We compared our results with the metrics reported by Ju and collaborators^18^ for a set of 30 molecules with experimental data, for which TD-DFT quantum chemical calculations were also performed. We applied our RF model to the same molecules and achieved metrics comparable to those of their quantum chemical calculations. The results can be seen in Table S10. We also performed predictions using our RF model on the complete dataset used by Ju and collaborators^18^ for training and testing. Our Random Forest model with their dataset and our training and test data splits achieved a mean absolute error (MAE) of 45.6 nm for the WLs and 0.22 for the QYs. Although these values are higher than those reported by Ju et al. (14.419 ± 0.683 nm and 0.11),^18^ they remain comparable to the TD-DFT results presented in the same study. This is particularly noteworthy, given that our models were not exposed to the underlying data trends during training.

Despite the strong performance of the ML models in the works of Joung et al. and Su et al.,^31,32^ they combined atomic structure data with additional features. In contrast, our approach relies solely on physicochemical and structural features extracted directly from RDKit. Furthermore, our work predicted the missing target features in the original Deep4Chem dataset using the RF model, ultimately creating two new and more complete datasets focused on WLs and QYs available for the scientific community.

### Shapley Additive Explanations (SHAP) Analysis

**Figure 5:**
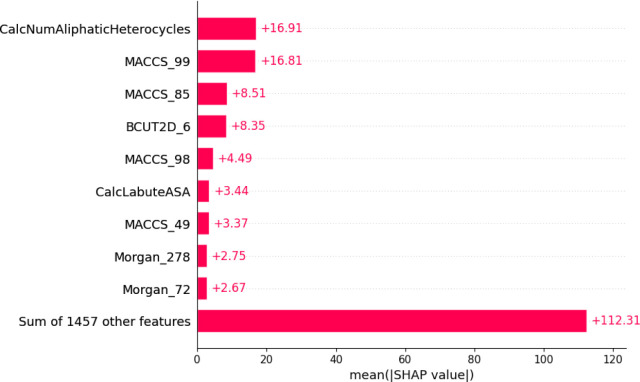
Plot of SHAP absolute values for the most important descriptors in predicting the emission wavelength target property prediction using the Random Forest model and the modified Deep4Chem database. Meaning of the descriptors: CalcNumAliphaticHeterocycles – number of aliphatic heterocyclic rings; MACCS_99 – C=C; MACCS_85 – CN(C)C; BCUT2D_6 – high Crippen MR eigenvalue (high Crippen MR contribution); MACCS_98 - six-membered heterocyclic ring; CalcLabuteASA – solvent accessible surface area calculated by Labute’s method; MACCS_49 – molecule containing at least one charged atom; Morgan_278 – [N]#C-*; and Morgan_72 – C(N(*)(*))(C*)C(*).

We applied SHAP analysis to the RF model by examining 300 randomly selected molecules, a number limited by the available computational resources. The fluorescence WL and QY target properties were investigated. Concerning the WLs, in Figures 5 and S4, the analysis revealed that the CalcNumAliphaticHeterocycles fingerprint property of the chromophore has the highest absolute value (16.9). This descriptor is the number of cyclic rings in a molecular structure that contains at least one noncarbon atom and is nonaromatic (i.e., heterocyclic rings). Cyclic rings influence the structural rigidity of a molecule, which, in turn, affects electronic conjugation. A more rigid structure typically results in more localized and well-defined electronic transitions instead of being delocalized across the molecule. This increased localization can enhance certain electronic states, potentially leading to fluorescence emission at more predictable and specific wavelengths.^47^

In our work on ML prediction of a molecule’s absorption energy, we also used the SHAP tool to analyze which descriptors had the greatest impact on the developed model.^29^ The third descriptor with the greatest impact on this absorption model was CalcNumAliphaticCarbocycles, with an impact of 0.18. This descriptor calculates the number of aliphatic cycles containing only the ring’s carbon atoms. Considering the light absorption and emission processes, there is a distinction in the favored descriptors: aliphatic heterocycles and aliphatic carbocycles contribute differently to the electronic properties of a molecule. While aliphatic heterocycles introduce heteroatoms that can participate in electronic interactions, increasing localized electronic transitions and structural rigidity (as observed for fluorescence WLs), aliphatic carbocycles lack such heteroatoms. Consequently, carbocycles contribute primarily to structural flexibility and delocalized electronic states, which can reduce predictability and shift absorption energies.^29^ The contrasting impacts of these descriptors highlight how including heteroatoms versus carbon-only rings influences molecular behavior differently depending on the target property (fluorescence wavelength or absorption energy).

Regarding the datasets used in this study, it is essential to note that all molecules in the two Deep4Chem-based datasets are organic chromophores. This ensures that the analysis focuses only on compounds with relevant emission properties. The second most impactful descriptor for the WLs in our RF model is MACCS_99 (absolute value equal to 16.49), related to the chromophore, which indicates a direct relationship between molecules containing the ethylene group (C=C) and fluorescence emission.^48^

**Figure 6:**
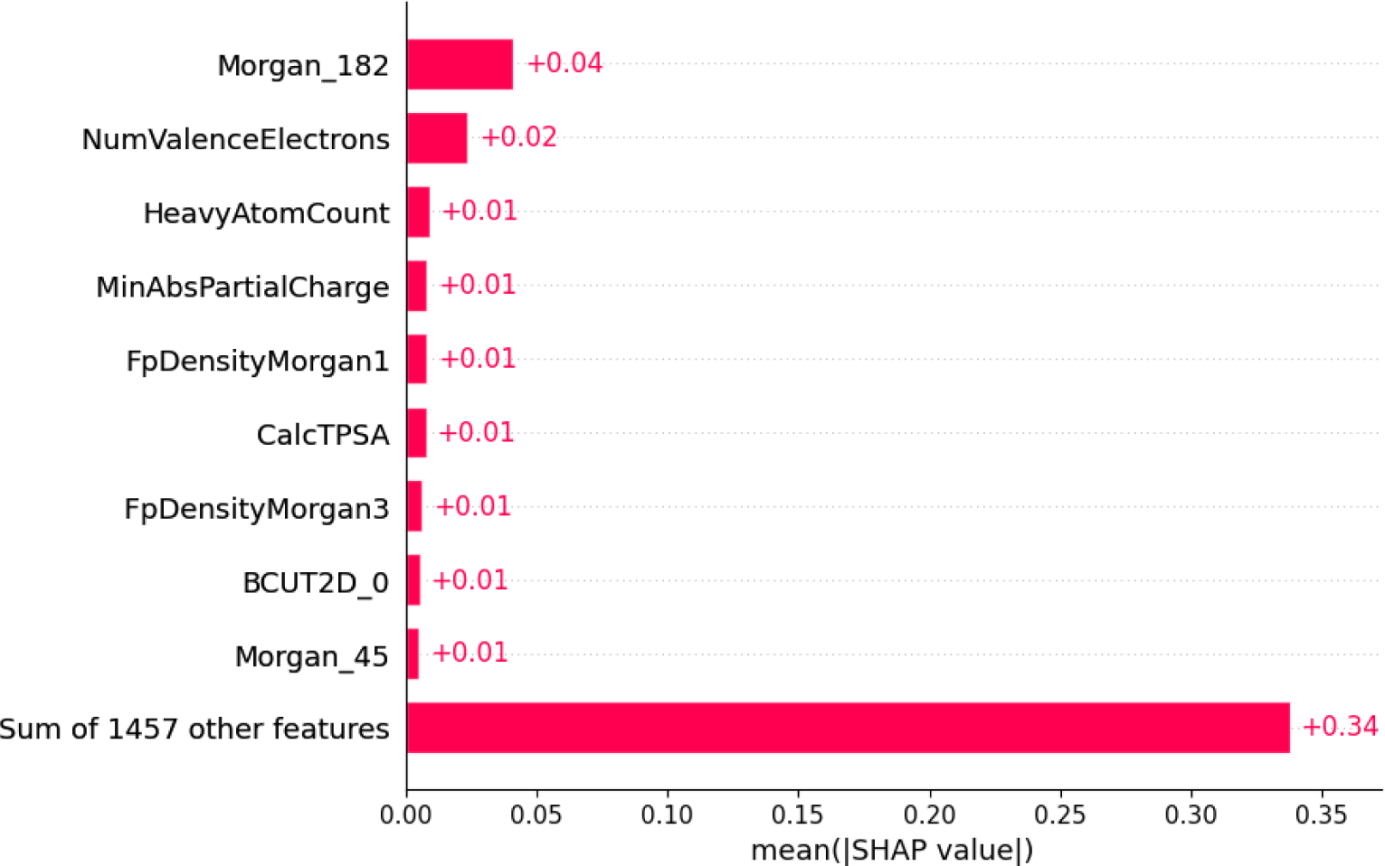
SHAP absolute values of the most important descriptors for the quantum yields prediction using Random Forest and the modified Deep4Chem database. Descriptors: Morgan_182 – central atom with one single and one double bond; NumValenceElectrons – number of valence electrons; HeavyAtomCount – number of heavy atoms in the molecular structure (excluding hydrogens); MinAbsPartialCharge – lowest absolute part load; FpDensityMorgan1 – Morgan fingerprint density of radius 1; CalcTPSA – calculated total polar surface area (TPSA) of the molecule in Å^2^ (square Angstroms); FpDensityMorgan3 – Morgan fingerprint density of radius 3; BCUT2D_0 – high mass eigenvalue, indicating that the molecule is composed of relatively heavy atoms or that the mass distribution in the molecule is such that the resulting eigenvalue is large; Morgan_45 – central atom bonded to 3 aromatic atoms in resonance, being two carbon atoms and one nitrogen atom.

**Figure 7:**
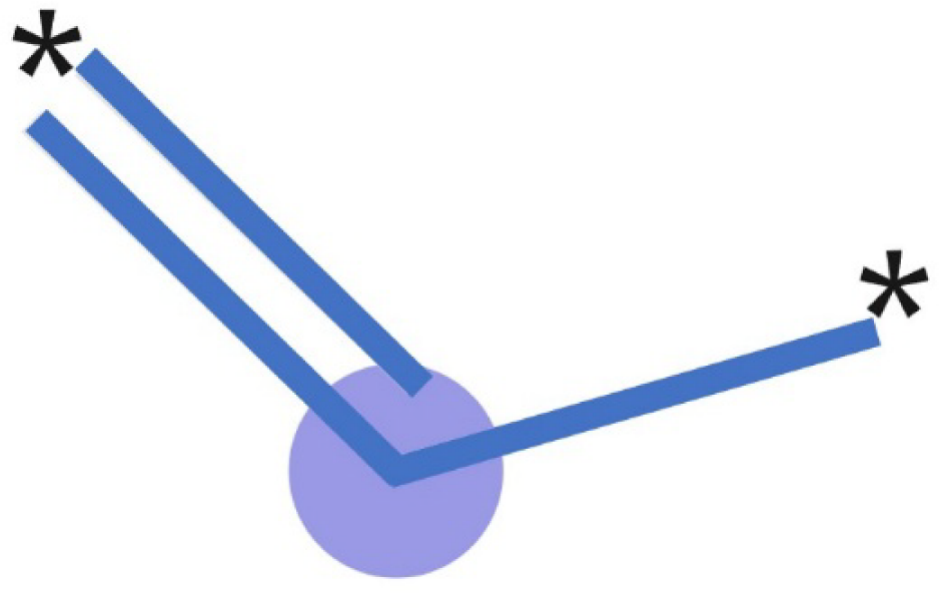
Fragment Morgan_182 – the central atom in the environment is represented in blue with one double bond and one single.

Regarding the RF model for the QYs, Morgan fingerprints emerged as the most significant from the SHAP analysis in Figures 6 and S5. The most critical in the chromophore is the Morgan_182 (absolute value equal to 0.04) fragment belonging to the molecular substructure depicted in Figure 7. A double bond within a fragment contributes to the electronic conjugation within the molecule, which is crucial for light absorption and emission.^49^ Just as this fragment was important for our previous ML absorption model, its absolute impact on the present work was 0.15.^29^ The more significant the molecular conjugation, the higher the likelihood of achieving a high quantum yield.^50^ Additionally, fluorine atoms, known for their high electronegativity, can extensively influence the molecule’s electronic distribution, potentially enhancing the stability of the excited state and increasing the likelihood of fluorescence emission.^51^

The chromophore’s NumValenceElectrons (absolute value equal to +0.02) descriptor is the second most important property for the QYs and represents the number of valence electrons. This descriptor is significant because valence electrons play a key role in molecular interactions, affecting reactivity and stability.^52^ Another impactful descriptor is the MinAbsPartialCharge, which indicates the minimum absolute partial charge in a molecule’s atoms. The partial charge of an atom measures the distribution of the electric charge around that atom. This charge distribution is essential during the excitation and emission of fluorescence, as the molecule undergoes electronic reorganization. If the partial charge distribution is highly polarized (i.e., the MinAbsPartialCharge value is considerable), the molecule experiences more significant electronic reorganization, increasing the likelihood of energy loss through nonradiative pathways, thereby reducing the quantum yield.^53^

While examining the two investigated properties, emission wavelengths (WLs) and fluorescence quantum yields (QYs), we observed that none of the top nine important SHAP descriptors were solvent-related. By evaluating our model directly with the ScikitLearn feature_importances function, we could determine the descriptor directly related to the solvent. This descriptor is the MinPartialCharge, which concerns the solvent for the WL target property. MinPartialCharge computes the minimum atomic partial charge within a molecule. Even though this descriptor related to the solvent is the most impactful, its value is 16 times lower than that of the most important descriptor concerning the chromophore.

The same approach was used to evaluate the solvent descriptor of greatest significance for the QY prediction model. The descriptors CalcKappa2 and MinAbsPartialCharge have nearly identical importance levels, around nine times lower than the most important descriptor associated with the chromophore. CalcKappa2 represents the second variant of Kier’s molecular flexibility index, while MinAbsPartialCharge, which is also relevant for predicting the WLs, was defined in the previous paragraph. It is important to note that their contributions here are also minimal, similar to those of the WL target property.

Despite the low importance of solvent-related descriptors in the SHAP analysis, a detailed investigation of prediction errors concerning the presence of specific descriptors revealed a direct correlation between solvent molecular fragments and the decreased model accuracy. The fragment linked to the highest prediction error was Morgan 443 (presence of one or more aromatic rings), found in 7.4% of our dataset. Further analysis indicated that this fragment in the solvent molecule decreases the prediction accuracy of the WL target property.

The second molecular fragment that most reduces the accuracy of the solvent modeling is Morgan 456 (the presence of an ether group), appearing in 6.07% of our dataset. Our analysis suggests that this fragment may introduce additional complexity and lead to model misclassification. Furthermore, Morgan 476 (connection between an ether group and an aromatic ring) appeared in 5.4% of our dataset among the chromophore-related fragments. This fragment indicates that such structural motifs may present significant challenges to the WL predictive accuracy in our Random Forest model.

For the QY target property, the fragment most frequently associated with prediction errors was also solvent-related, specifically Morgan 398 (connection between two aromatic rings, where one of the rings contains both a nitrogen and a sulfur atom) present in 6.5% of our dataset. The fragment that contributed most to errors for the QY predictions was Morgan 324 (naphthalate group presence), present in 6.4% of the molecules in our dataset. Our findings suggest that this fragment introduces additional complexity, increasing model misclassification.

To sum up, the ML models developed here emphasize the chromophore itself, which aligns with chemical intuition: the chromophore molecule dictates the presence or absence of emission, whereas the solvent affects its intensity and position.

### ML-Generated Databases

**Figure 8:**
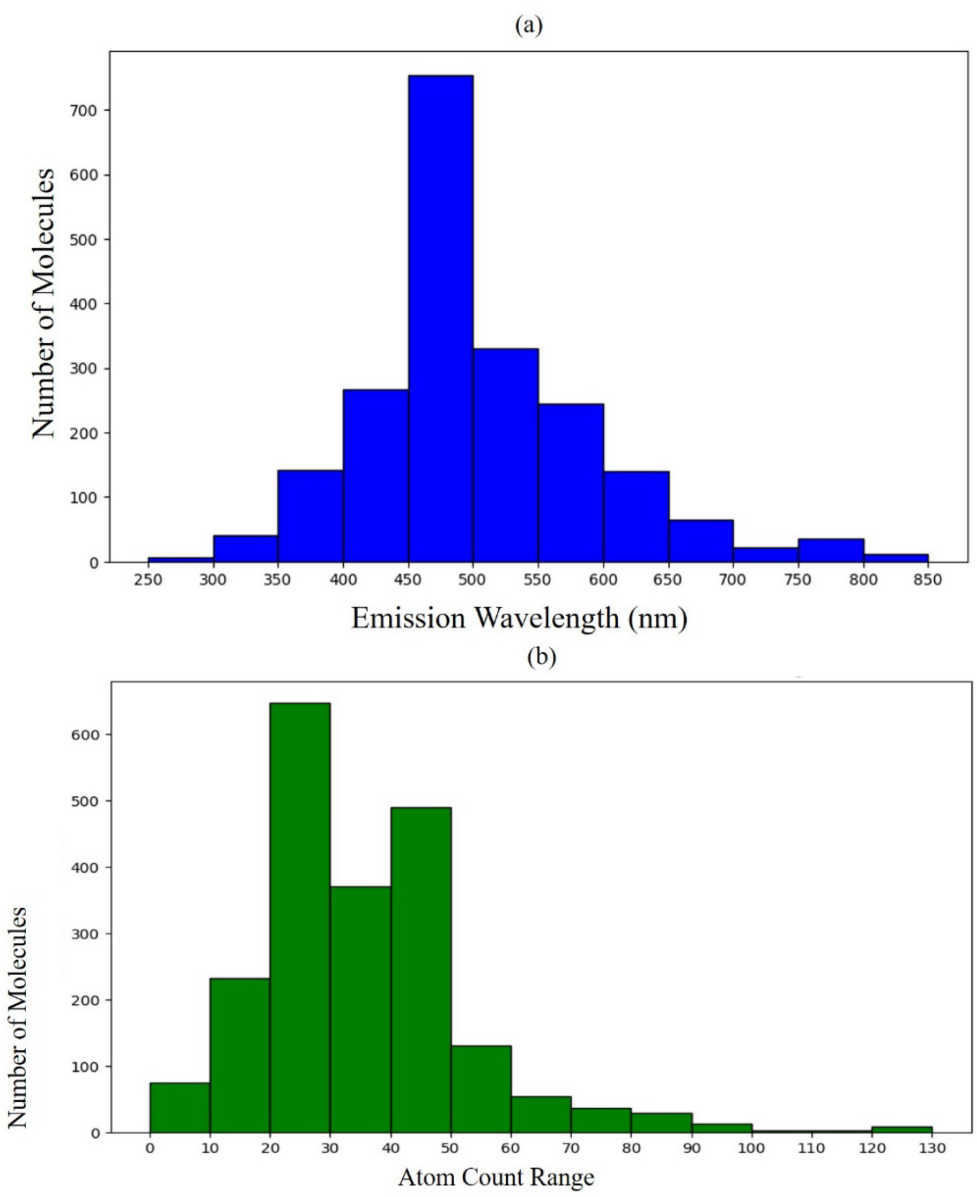
(a) Distribution of molecules by emission wavelengths of the data computed in this work; (b) distribution of molecules by the number of atoms of the data computed in this work.

The RF ML models were used to compute the WL and QY missing data from the original Deep4Chem dataset. Thus, using the 2,094 combinations of molecules and solvents in our RF model, a database containing 2,056 combinations for the WL property was computed after validating SMILES and data cleaning. The 38 combinations of molecules (chromophores and solvents) that could not be converted with RDKit were eliminated from the final set. The distribution of these produced data is shown in Figure 8a. Figure 8b displays the average distribution of the number of atoms in this newly created database, including the predicted properties. A comparison between the molecules in the available data and those with missing data reveals a similarity that provides confidence in the predicted data from our RF model.

**Figure 9:**
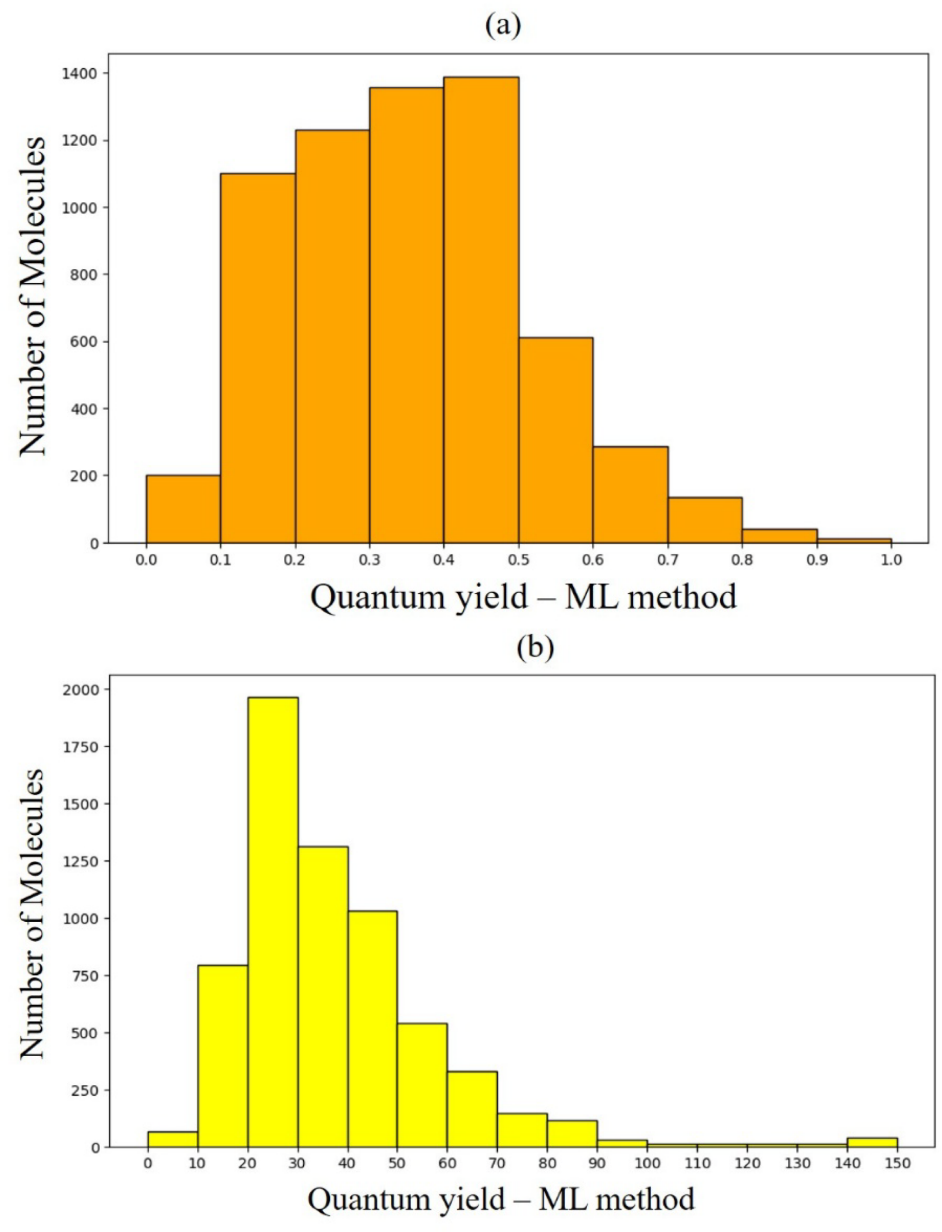
(a) Distribution of molecules according to their quantum yields; (b) distribution of molecules by the number of atoms.

The RF model computed missing QYs, generating a database of 6,360 validated chromophore–solvent combinations with QY properties from 6,399 initial combinations after SMILES validation and data cleaning. A total of 39 combinations of molecules and solvents could not be converted with RDKit, so they were eliminated from the final set. The distribution of the computed data is shown in Figure 9a. Figure 9b displays the average distribution of the number of atoms in this newly created database for the predicted target properties. Similar to the computed WL data, the distributions of the QY properties also show a high degree of similarity to the data distribution from the original database, providing increased confidence in the predictions.

### Fluorescence Prediction

**Figure 10:**
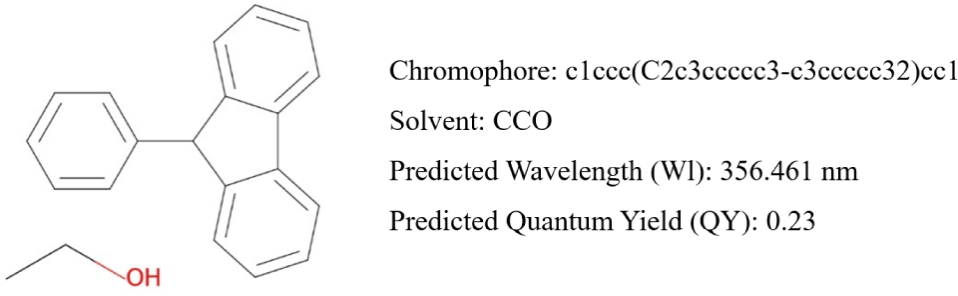
Example of a complete forecast using the developed RF models.

The code for predicting the emission wavelengths and quantum yield is available in the project’s GitHub and Zenodo repositories.^44^ Users need to provide only a SMILES representation for the chromophore and another for the solvent to run it. The RF model then predicts the WL and QY target properties, as illustrated in Figure 10. The figure shows a molecule from the dataset used to train and validate the RF models.

We selected 10 fluorophores and their respective solvents from the PhotochemCAD database to evaluate our models.^54^ We calculated the error between the predictions with our RF model and the experimental values obtained from the PhotochemCAD database, yielding an RMSE of 45.09 nm for WLs and an RMSE of 0.15 for QYs. The RMSE for the WL target property shows a slight increase compared to the values of our test set, which may be attributed to the fact that none of the selected molecules was previously seen during the training of the RF model. In contrast, the RMSE for the QY target property was lower than that observed for our test set. This suggests that the RF model for QYs is more robust and can generalize well to data outside of the similarity with unseen data. At the same time, for WLs, the same degree of generalization does not occur. The data used are also available on the GitHub repository.

## Conclusions

We investigated different ML models to predict essential photophysical properties of molecules related to fluorescence, namely, fluorescent emission wavelengths (WLs) and quantum yields (QYs). For this purpose, we used the experimental optical database Deep4Chem built by Joung and coworkers.^30^ For each molecule in the 20,236 set, 18,142 combinations include the WL target property, and 13,837 combinations include the QY target property. We used two SMILES (chromophore + solvent) for each entry in the Deep4Chem database, the first one for the chromophore molecule and the other for the solvent, to generate, using the RDKit tool, 1,466 descriptors and fingerprints (512 Morgan “chromophore”, 512 Morgan “solvent”, 167 MACCs “chromophore”, 167 MACCs “solvent”, 54 chemical descriptors “chromophore”, and 54 chemical descriptors “solvent”) to be employed in different ML models. By computing different error metrics, we selected three machine learning algorithms (ExtraTrees, Random Forest, and XGB), and we also developed a neural network that predicts the two properties for a test set comprising 30% of the total number of entries in Deep4Chem. The best prediction of the two target properties was achieved with the RF algorithm.

The results of the Random Forest models applied to the test set demonstrated good generalization, because they presented very similar metrics between the validation sets. However, these metrics were slightly worse compared to other machine learning models like the graph convolutional network (GCN)^31^ and message-passing neural network (MPNN)^32^ models for WLs that used molecular structures as inputs for predicting fluorescence emission wavelengths. The same RF models were used to calculate the QY target property, and our RF model presented better metrics than the GCN and metrics very close to those of the MPNN, using very different types of inputs.

SHAP analysis indicated that the descriptor CalcNumAliphaticHeterocycles holds the highest absolute importance to the WL model RF, and Fragment Morgan_182, which represents a central double bond and a single bond, has the highest absolute importance for the QY model RF.

With our RF models, we created two new databases from the Deep4Chem database, filling in the missing information.^30^ The first one contains the SMILES (chromophore + solvent) and the WL property, while the other contains the SMILES and QY property. Additionally, we used our RF models to predict these two properties, which we implemented in a tool available in the project’s GitHub and Zenodo repositories.^44^ By providing two molecules in the SMILES format, one representing the chromophore and the other the solvent, it is possible to predict the two target properties using the tool. Our tests showed that our RF models achieved an RMSE of 45.17 nm for WL and 0.148 for QY when applied to unseen fluorophores and solvents obtained from the PhotochemCAD database, thus displaying strong generalization for QY predictions and a slightly higher error for WLs. These results highlight the model’s potential for accurate property prediction even with new molecular combinations.

## Data Availability

The source code of this work, machine learning model parameters, input files, SHAP values, and output examples are available in the laboratory repository, accessible at: https://github.com/Quimica-Teorica-IME and on Zenodo.^44^

## Supporting Information Available

The distribution of database values, a description of employed descriptors and metrics, detailed results obtained with the developed models, learning curves, and SHAP metrics (PDF)
