# Supplementary material for: Predicting Fluorescence Emission Wavelengths and Quantum Yields via Machine Learning
Source: J Chem Inf Model. 2025 Mar 20;65(7):3270–81. doi: 10.1021/acs.jcim.4c02403 (PMC12004507; doi:10.1021/acs.jcim.4c02403)
Supplement: Supplementary file 1 — ci4c02403_si_001.pdf [file ci4c02403_si_001.pdf]

Supporting Information:

Predicting Fluorescence Emission Wavelengths and Quantum Yields via Machine Learning

Rubens C. Souza,[a,1] *Julio C. Duarte,*[a,b,2] *Ronaldo R. Goldschmidt,*[a,b,3] *Itamar Borges, Jr..*[a,c,4,*]

[a] Departamento de Engenharia de Defesa, Instituto Militar de Engenharia (IME), Praça Gen. Tibúrcio 80, Rio de Janeiro, RJ, 22290 270, Brazil

[b] Departamento de Engenharia da Computação, Instituto Militar de Engenharia (IME), Praça Gen. Tibúrcio 80, Rio de Janeiro, RJ, 22290 270, Brazil

[c] Departamento de Química, Instituto Militar de Engenharia (IME), Praça Gen. Tibúrcio 80, Rio de Janeiro, RJ, 22290 270, Brazil

** E-mail: itamar@ime.eb.br*

[1] *https://orcid.org/0009-0006-7597-6464*

[2] *https://orcid.org/0000-0001-6656-1247*

[3] *https://orcid.org/0000-0003-1688-0586*

[4] *http://orcid.org/0000-0002-8492-1223*

1. MOLECULE DATABASE

Figure S1 depicts the data distribution in the Deep4Chem database.[1]

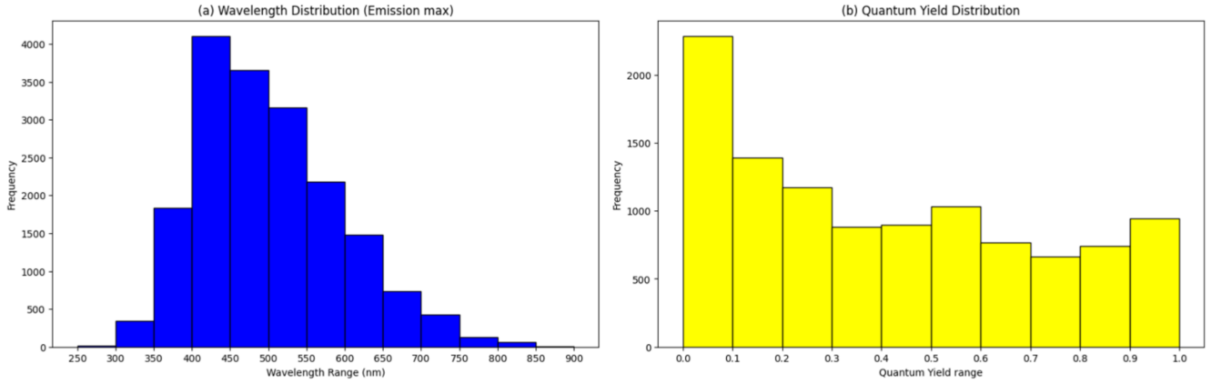

Figure S1. Distribution of molecular properties from Deep4Chem. (a) Distribution of wavelengths in nm; (b) Distribution of quantum yield values.

2. RDKIT DESCRIPTORS

Table S1 lists all 54 descriptors obtained from RDKit,[2] which were used as part of the descriptors to develop all the ML models in this work. All descriptors found in RDKit that could be used with a SMILES format were used. The MACCS (Molecular ACCess System) fingerprints obtained from RDKit are a type of structural key used in cheminformatics to encode molecular features into a binary vector, which serves as a compact representation of a molecule's structure.[3] The MORGAN fingerprints from RDKit, also known as circular fingerprints, are a class of molecular descriptors widely used in cheminformatics for encoding the structural features of molecules into a binary or a count vector based on the Extended-Connectivity Fingerprints (ECFP) algorithm.[4] Morgan fingerprints systematically capture the presence of atom-centered circular substructures of varying radii and are detailed by RDKit.[5]

Table S1. Fingerprints and descriptors used to develop the machine learning models.

Descriptors	Description
FpDensityMorgan1	Morgan fingerprint density of radius 1
FpDensityMorgan2	Morgan fingerprint density of radius 2
FpDensityMorgan3	Morgan fingerprint density of radius 3
ExactMolWt	Exact molecular weight
HeavyAtomMolWt	Molecular weight considering only heavy atoms
MaxAbsPartialCharge	Largest absolute partial load
MaxPartialCharge	Largest partial load
MinAbsPartialCharge	Lowest absolute part load
MinPartialCharge	Lower partial load
NumRadicalElectrons	Number of radical electrons
NumValenceElectrons	Number of valence electrons
CalcFractionCSP3	Fraction of carbon atoms that are sp^3
CalcKappa1	First form of Kier's molecular flexibility index
CalcKappa2	Second form of Kier's molecular flexibility index
CalcKappa3	Third form of Kier's molecular flexibility index
CalcLabuteASA	Solvent accessible surface area calculated by Labute's method
CalcNumAliphaticCarbocycles	Number of aliphatic carbocyclic rings
CalcNumAliphaticHeterocycles	Number of aliphatic heterocyclic rings
CalcNumAliphaticRings	Total number of aliphatic rings
CalcNumAmideBonds	Number of amide bonds
CalcNumAromaticCarbocycles	Number of aromatic carbocyclic rings
CalcNumAromaticHeterocycles	Number of aromatic heterocyclic rings
CalcNumAromaticRings	Total number of aromatic rings
CalcNumAtomStereoCenters	Number of atomic stereogenic centers
CalcNumBridgeheadAtoms	Number of bridging atoms in bicyclic structures
CalcNumHBA	Number of hydrogen bond acceptor atoms
CalcNumHBD	Number of hydrogen bond donor atoms
CalcNumHeteroatoms	Number of heteroatomic atoms (non-carbon and non-hydrogen)
CalcNumHeterocycles	Number of heterocyclic rings
CalcNumLipinskiHBA	Number of hydrogen bond acceptors according to Lipinski's rule
CalcNumLipinskiHBD	Calculated number of hydrogen bond donors according to Lipinski's rule
CalcNumRings	Total number of rings in the molecular structure
CalcNumRotatableBonds	Number of rotatable bonds in the molecule
CalcNumSaturatedCarbocycles	Number of saturated carbocycles in the molecule
CalcNumSaturatedHeterocycles	Number of saturated heterocycles in the molecule
CalcNumSaturatedRings	Number of saturated rings in the molecule
CalcNumSpiroAtoms	Number of spiro atoms (bonding atoms between two rings) in the molecule
CalcNumUnspecifiedAtomStereoCenters	Number of stereoisomeric centers of unspecified atoms in the molecule

CalcTPSA	Calculated total polar surface area (TPSA) of the molecule in Å² (square Angstroms)
HeavyAtomCount	Number of heavy atoms in the molecular structure (excluding hydrogens)
NHOHCount	Number of N-H and O-H groups in the molecule
NOCount	Total number of nitrogen and oxygen atoms in the molecule
NumHAcceptors	Number of hydrogen atoms acting as H-bond acceptors
NumHDonors	Number of hydrogen atoms acting as H-bond donors
Chi0	Chi0 connectivity index
Chi1	Chi1 connectivity index
BCUT2D_MW_Eigval0	High mass eigenvalue, indicating that the molecule is composed of relatively heavy atoms or that the mass distribution in the molecule is such that the resulting eigenvalue is large
BCUT2D_MW_Eigval1	Low mass eigenvalue suggests that the molecule is composed of relatively light atoms or that the mass distribution in the molecule is such that the resulting eigenvalue is small
BCUT2D_Chg_Eigval0	High Gasteiger charge eigenvalue (high contribution from Gasteiger charge)
BCUT2D_Chg_Eigval1	Low Gasteiger charge eigenvalue (low contribution from Gasteiger charge)
BCUT2D_CrippenLogP_Eigval0	High Crippen logP eigenvalue (high contribution from Crippen logP)
BCUT2D_CrippenLogP_Eigval1	Low Crippen logP eigenvalue (low contribution from Crippen logP)
BCUT2D_CrippenMR_Eigval0	High Crippen MR eigenvalue (high Crippen MR contribution)
BCUT2D_CrippenMR_Eigval1	Low Crippen MR eigenvalue (low Crippen MR contribution)

3. VALIDATION METRICS OF THE MACHINE LEARNING ALGORITHMS

The Root Mean Square Error (RMSE) value is an error metric widely used in machine learning to compare and evaluate regression models.[6] In particular, very low RMSE values in the training set may indicate model overfitting. The RMSE is defined as

$$RMSE = \sqrt{\frac{1}{n}\sum_{i=1}^{n}(y_i - \bar{y}_i)^2} \qquad (1)$$

where n represents the total number of samples, y is the observed value, and $\bar{y}$ is the value predicted by the algorithm. In our case, y represents the DFT and TD-DFT computed properties extracted from the original QM-symex dataset, while $\bar{y}$ is the value predicted by our machine learning (ML) models. The squared difference $(y - \bar{y})^2$ penalizes larger deviations more severely, particularly significant outliers. The square of the RMSE is the Mean Squared Error (MSE) or variance.[7] The Mean Absolute Error (MAE), another useful error metric, is conventionally defined as $\frac{1}{n}\sum_{i=1}^{n}|y - \bar{y}|$.[6]

The coefficient of determination R^2 is a metric that measures the proportion of variability in the predicted values by a ML model. It is a useful statistic quantity for assessing how well the model fits the data. R^2 ranges from 0 to 1, where 1 indicates a perfect fit of the model to the data. An R^2 value close to 1 means that a large proportion of the variability in the data is explained by the ML model.[8] Conversely, when this value is close to 0, it suggests that the model does not adequately describe the variability in the data. The R^2 metric is defined as:

$$R^2 = 1 - \frac{\sum_{i=1}^{n}(y_i - \bar{y}_i)^2}{\sum_{i=1}^{n}(y_i - \tilde{y}_i)^2} \qquad (2)$$

where n is the total number of samples, y is the observed value, $\bar{y}$ is the value estimated by the algorithm, and $\tilde{y}$ is the mean of the observed values.

4. LAZY PREDICT AND COMPARISON OF ALGORITHMS

Tables S2 and S3 present the results of the Lazy Predict tool for the ML models developed in this work for predicting the emission wavelength and quantum yield target properties. Tables S8 to S11 show the results of the three most frequent top-performing ML models identified by Lazy Predict and an additional model, a neural network, with the respective error metrics. Note that there is a difference between ExtraTreeRegressor and ExtraTreesRegressor. The ExtraTreesRegressor (Extremely Randomized Trees Regressor)[9] is an ensemble method that aggregates multiple decision trees, whereas the ExtraTreeRegressor (Extra Tree Regressor)[10] is a single decision tree without an ensemble approach.

Table S2. Table S. Lazy Predict results for the emission wavelength target property. Refer to the discussion above about the difference between the ExtraTreeRegressor and the ExtraTreesRegressor ML models.

Model	R-Squared	RMSE (nm)
ExtraTreesRegressor	0.92	26.69
RandomForestRegressor	0.90	29.23
XGBRegressor	0.89	30.98
BaggingRegressor	0.89	31.89
LGBMRegressor	0.86	35.58
HistGradientBoostingRegressor	0.86	35.81
MLPRegressor	0.84	37.63
ExtraTreeRegressor	0.81	40.68
DecisionTreeRegressor	0.79	43.63
KNeighborsRegressor	0.73	48.70
GradientBoostingRegressor	0.71	50.82
LassoCV	0.67	54.08
PoissonRegressor	0.67	54.18
BayesianRidge	0.67	54.42
RidgeCV	0.67	54.42
Ridge	0.67	54.66
HuberRegressor	0.66	54.98
ElasticNetCV	0.66	55.24
LinearSVR	0.66	55.32
OrthogonalMatchingPursuitCV	0.62	58.20
OrthogonalMatchingPursuit	0.62	58.20
Lasso	0.61	58.69
ElasticNet	0.59	60.90
GammaRegressor	0.57	61.72
TweedieRegressor	0.57	61.87
LassoLars	0.56	62.73
LassoLarsIC	0.56	62.73
LassoLarsCV	0.56	62.73

AdaBoostRegressor	0.46	69.44
SVR	0.39	74.13
NuSVR	0.36	75.48
LarsCV	0.27	80.67
PassiveAggressiveRegressor	0.18	85.61
DummyRegressor	-0.00	94.55
GaussianProcessRegressor	-26.78	498.34
KernelRidge	-27.29	502.85
RANSACRegressor	-213359262593949600.00	43672566173.44
LinearRegression	-1542758546107555584.00	117436186814.74
TransformedTargetRegressor	-1542758546107555584.00	117436186814.74
SGDRegressor	-10882554929657665536.00	311902168840.21
Lars	-1,47209E+29	3,62761E+16

Table S3. Lazy Predict results for the quantum yield

Model	R-Squared	RMSE
ExtraTreesRegressor	0.65	0.18
RandomForestRegressor	0.63	0.19
XGBRegressor	0.59	0.20
BaggingRegressor	0.57	0.21
NuSVR	0.54	0.21
SVR	0.54	0.21
LGBMRegressor	0.53	0.21
HistGradientBoostingRegressor	0.52	0.22
MLPRegressor	0.44	0.23
KNeighborsRegressor	0.40	0.24
GradientBoostingRegressor	0.31	0.26
DecisionTreeRegressor	0.30	0.26
ExtraTreeRegressor	0.29	0.26
LassoCV	0.27	0.27
ElasticNetCV	0.27	0.27
BayesianRidge	0.27	0.27
RidgeCV	0.26	0.27
HuberRegressor	0.25	0.27
Ridge	0.23	0.27
OrthogonalMatchingPursuit	0.22	0.28
OrthogonalMatchingPursuitCV	0.22	0.28
TweedieRegressor	0.20	0.28
GammaRegressor	0.20	0.28
PoissonRegressor	0.16	0.28
LassoLarsCV	0.16	0.29
LassoLarsIC	0.16	0.29
AdaBoostRegressor	0.10	0.30
LarsCV	0.02	0.31
Lasso	-0.00	0.31

ElasticNet	-0.00	0.31
DummyRegressor	-0.00	0.31
LassoLars	-0.00	0.31
LinearSVR	-0.19	0.34
PassiveAggressiveRegressor	-0.37	0.36
RANSACRegressor	-0.57	0.39
KernelRidge	-1.08	0.45
GaussianProcessRegressor	-1.19	0.46
Lars	-517549876638516.31	7087022.84
LinearRegression	-16083782949256968192.00	1249343406.86
TransformedTargetRegressor	-16083782949256968192.00	1249343406.86
SGDRegressor	-2716661600678908971188224.00	513458679420.73

In Tables S4 to S7, the values highlighted in bold represent the best error metrics for a given ML model, with the Random Forest consistently demonstrating the superior metrics among those we investigated.

Table S4. Mean Absolute Error (MAE) values for each of the ML models applied to the test set

Property	Extra trees	Random forest	XGB	RNA
WaveLen (nm)	19.5	**18.56**	84.1	30.8
Quantumyield	0.16	**0.14**	0.26	4.21

Table S5.Mean Squared Error (MSE) values for each of the ML models applied to the test set

Property	Extra trees	Random forest	XGB	RNA
WaveLen (nm)	906.2	**828.62**	9773.63	2062.89
Quantumyield	0.05	**0.04**	0.10	59.13

Table S6. Root Mean Square Error (RMSE) values for each of the ML models applied to the test set

Property	Extra trees	Random forest	XGB	RNA
WaveLen (nm)	30.10	**28.78**	98.86	45.41
Quantumyield	0.22	**0.19**	0.32	7.68

Table S7. R^2 values for each of the ML models applied to the test set

Property	Extra trees	Random forest	XGB	RNA
WaveLen (nm)	0.89	**0.90**	-0.12	0.76
Quantumyield	0.51	**0.62**	-0.09	-614.19

5. CROSS-VALIDATION

Random Forest model cross-validation results, which achieved the best metrics, are presented in Tables S8 and S9 and the learning curves in Figures S2 and S3. The cross-validation results for the other models can be found in our GitHub.

Table S8. Cross-validation results for the emission wavelength in the Random Forest model

Folds	MAE	MSE	RMSE	R^2
Fold 1	22.345	1102.07	33.197	0.87
Fold 2	22.998	1183.50	34.402	0.87
Fold 3	22.762	1193.597	34.548	0.86

Table S9. Cross-validation results for the Quantum yield in the Random Forest model

Folds	MAE	MSE	RMSE	R^2
Fold 1	0.156	0.044	0.21	0.54
Fold 2	0.159	0.044	0.21	0.55
Fold 3	0.157	0.044	0.21	0.54

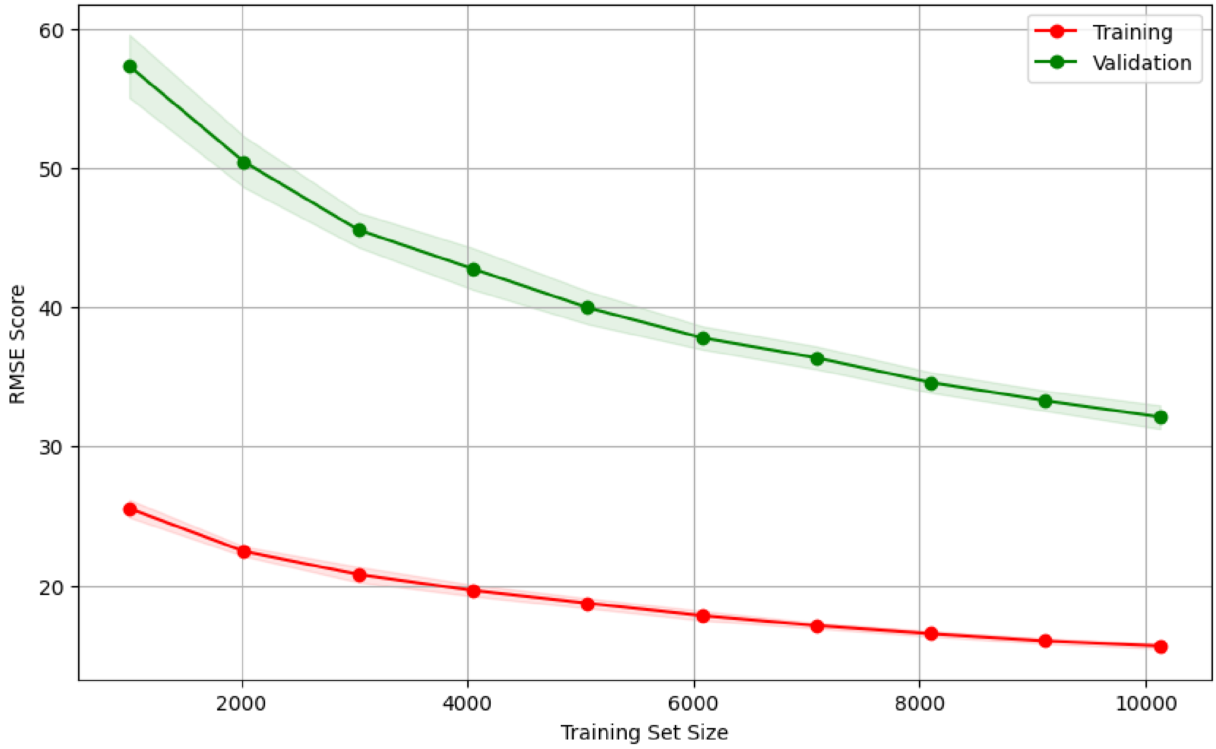

Figure S2. Graph of the learning curve of the Random Forest model with RMSE score for the Emission Wavelength property.

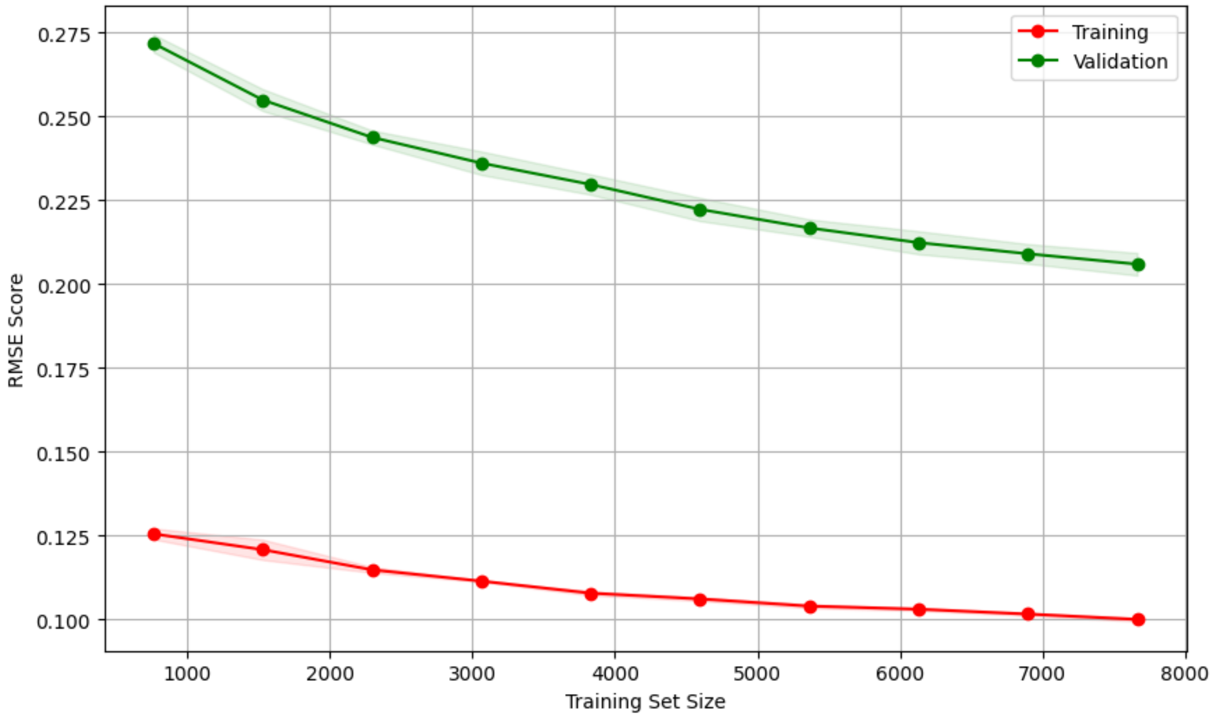

Figure S3. Graph of the learning curve of the Random Forest model with RMSE score for the Quantum yield property.

6. SHAP METRICS

The plots below depict the SHapley Additive ExPlanations (SHAP) absolute values of 10 features that have the most significant impact on the best prediction model, the Random Forest, for each target property (WL, QY). The plots for the other models, illustrated in Figures S3 to S12, are available on our GitHub.

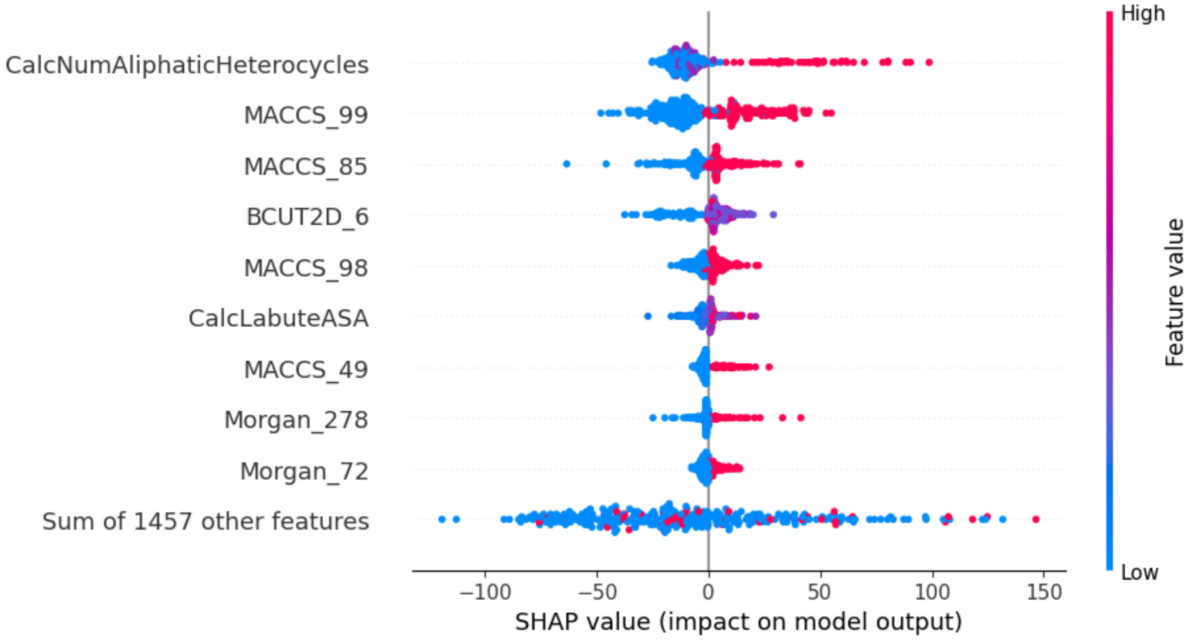

Figure S4. Beeswarm plot of the Random Forest model for the emission wavelength target property.

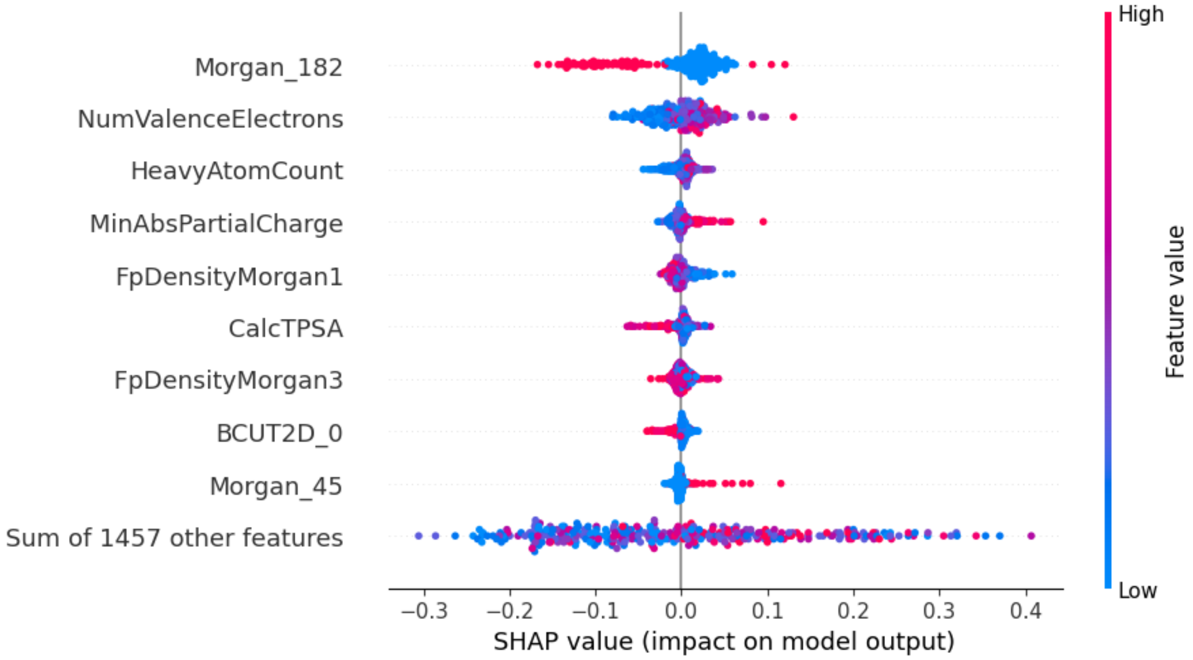

Figure S5. Beeswarm plot of the Random Forest model for the quantum yield.

7. Comparison with literature

Table S10 presents the MAE values for the 30 molecules from the study by Ju and collaborators,[11] which utilized quantum chemical computational methods (DFT with the wB97xd functional) and machine learning techniques (Gradient Boosting Regression Tree – GBRT).

Table S10. MAE result for the emission (WL) wavelength (nm/eV) and (QY) Quantum Yield prediction of each model.

Property	RF (present)	GBRT[11]	wB97xd[11]
Wavelenghts (nm)	37 nm/ 0.16 eV	23 nm/ 0.09 eV	57 nm/ 0.26 eV
Quantum yields	0.27	0.20	-

Supplementary data

The source code of this work, machine learning model parameters, input files, SHAP values, and output examples are available in the laboratory repository, accessible at: https://github.com/Quimica-Teorica-IME , and at Zenodo.[12]

REFERENCES

(1) Joung, J. F.; Han, M.; Jeong, M.; Park, S. Experimental Database of Optical Properties of Organic Compounds. *Sci. Data* **2020**, *7* (1), 295. DOI: https://doi.org/10.1038/s41597-020-00634-8.

(2) LANDRUM, G. Rdkit documentation, 2019. https://readthedocs.org/projects/rdkit/downloads/pdf/latest/ (accessed 2024-10-22).

(3) LANDRUM, G. RDKit: MACCS Keys Module. 2024. https://github.com/rdkit/rdkit/blob/master/rdkit/Chem/MACCSkeys.py (accessed 2024-07-02).

(4) Rogers, D.; Hahn, M. Extended-Connectivity Fingerprints. *J. Chem. Inf. Model.* **2010**, *50* (5), 742–754. DOI: https://doi.org/10.1021/ci100050t.

(5) RDKit. Getting Started in Python: Morgan Fingerprints. 2024. https://www.rdkit.org/docs/GettingStartedInPython.html#morgan-fingerprints (accessed 2024-07-02).

(6) Hodson, T. O. Root-Mean-Square Error (RMSE) or Mean Absolute Error (MAE): When to Use Them or Not. *Geoscientific Model Development*. Copernicus GmbH July 19, 2022, pp 5481–5487. DOI: https://doi.org/10.5194/gmd-15-5481-2022.

(7) Qi, J.; Du, J.; Siniscalchi, S. M.; Ma, X.; Lee, C. H. On Mean Absolute Error for Deep Neural Network Based Vector-to-Vector Regression. *IEEE Signal Process. Lett.* **2020**, *27*, 1485–1489. DOI: https://doi.org/10.1109/LSP.2020.3016837.

(8) Balal, A.; Pakzad Jafarabadi, Y.; Demir, A.; Igene, M.; Giesselmann, M.; Bayne, S. Forecasting Solar Power Generation Utilizing Machine Learning Models in Lubbock. *Emerging Science Journal* **2023**, *7* (4), 1052–1062. DOI: https://doi.org/10.28991/ESJ-2023-07-04-02.

(9) Geurts, P., Ernst, D. & Wehenkel, L. Extremely randomized trees. *Mach. Learn.* **2006**, *63*, 3–42. DOI: https://doi.org/10.1007/s10994-006-6226-1

(10) ScikitLearn. ExtraTreeRegressor. 2025. https://www.rdkit.org/docs/GettingStartedInPython.html#morgan-fingerprints (accessed 2025-02-12).

(11) Ju, C. W.; Bai, H.; Li, B.; Liu, R. Machine Learning Enables Highly Accurate Predictions of Photophysical Properties of Organic Fluorescent Materials: Emission Wavelengths and Quantum Yields. *J. Chem. Inf. Model.* **2021**, *61* (3), 1053–1065. DOI: https://doi.org/10.1021/acs.jcim.0c01203.

(12) Souza, R.; Duarte, J.; Goldschmidt, R.; Borges Jr, I. Predicting Fluorescence Emission Wavelengths and Quantum Yields via Machine Learning. *Zenodo* **2025**. DOI: https://doi.org/10.5281/zenodo.14826586.
